# Supplementary material for: Airway epithelium respiratory illnesses and allergy (AERIAL) birth cohort: study protocol
Source: Front Allergy. 2024 Apr 11;5:1349741. doi: 10.3389/falgy.2024.1349741 (PMC11043573; doi:10.3389/falgy.2024.1349741)
Supplement: Supplementary file 1 [file Table1.docx]

**Supplementary Table 1:** List of all symptoms and medications included in the TempTracker app. Respiratory symptoms for case definition denoted with *.

| **SYMPTOMS** | **MEDICATIONS** |
| --- | --- |
| NONE | NONE |
| Nose – Runny* | Panadol |
| Nose – Blocked/stuffy* | Neurofen |
| Cough – Dry* | Ventolin |
| Cough – Wet* | Antibiotics |
| Sneezing* | Antihistamines |
| Headache* | Nasal Decongestant |
| Muscle Aches* | Cough syrup |
| Chills* | Steroids |
| Repeated shaking with chills* | Other -> Free Text Entry |
| Tiredness* |  |
| Sore Throat* |  |
| Having trouble breathing* |  |
| New loss of taste* |  |
| New loss of smells* |  |
| Feeding – not wanting* |  |
| Not eating solids* |  |
| Diarrhoea |  |
| Crying |  |
| Sleeping more |  |
| Feeds – wanting more |  |
| Cling |  |
| Dribbling |  |
| Rubbing at gums |  |
| Pulling at ears |  |
| Vomiting |  |
| Nappies – less wet |  |
| Nappies – more wet |  |
| Rash |  |
| Flushed cheeks/face |  |
| Wind/colic |  |
| Other -> Free Text Entry |  |
